# Distinct mechanisms of electroacupuncture and manual acupuncture in modulating hypothalamic GnRH–tanycyte unit function of polycystic ovary syndrome

**DOI:** 10.1186/s13020-025-01068-3

**Published:** 2025-02-05

**Authors:** Yu Wang, Yicong Wang, Yuning Chen, Wenhan Lu, Xiaoyu Tong, Jiajia Li, Wenhao Gao, Rui Huang, Wei Hu, Yi Feng

**Affiliations:** 1 Department of Integrative Medicine and Neurobiology, School of Basic Medical Sciences, State Key Laboratory of Medical Neurobiology and MOE Frontiers Center for Brain Science, Institutes of Brain Science , Fudan University, Shanghai, 200032 China; 2 Shanghai Key Laboratory of Acupuncture Mechanism and Acupoint Function, Shanghai Institute of Acupuncture and Moxibustion, Fudan University, Shanghai, 200433 China; 3 Department of Ophthalmology & Visual Science, Eye & ENT Hospital, Shanghai Medical College, Fudan University, Shanghai, China

**Keywords:** Polycystic ovary syndrome, Electroacupuncture, Manual acupuncture, GnRH–tanycyte unit, Itgb1

## Abstract

**Background:**

Polycystic ovary syndrome (PCOS) is a complex neuroendocrine disorder characterized by dysregulation of the hypothalamus. Both electroacupuncture (EA) and manual acupuncture (MA) have demonstrated therapeutic efficacy in the treatment of PCOS through improvements in hypothalamic function. However, the underlying mechanisms remain poorly understood. Gonadotropin-releasing hormone (GnRH) neurons are pivotal in regulating hypothalamic endocrine function, whereas tanycyte, a specialized glial cell type, potentially contribute to this process.

**Methods:**

A dihydrotestosterone (DHT)-induced PCOS-like mouse model was used to investigate the effects of acupuncture. Tissue clearing and three-dimensional (3D) imaging were employed to visualize the hypothalamic GnRH neuronal network and assess postacupuncture modifications. Transcriptome sequencing was performed to identify changes in the gene profiles associated with EA and MA. Rax-CreER^T2^ transgenic mice were utilized to investigate the molecular targets of EA in tanycytes.

**Results:**

EA significantly alleviated neuroendocrine dysfunction in PCOS-like mice by restoring the density and coverage of GnRH axonal projections. MA displayed similar therapeutic effects but had less pronounced effects on GnRH axons. Transcriptome analysis revealed distinct mechanisms for these two approaches: EA primarily regulates neuroglial plasticity, whereas MA predominantly targets neurotransmitter regulation. Both EA and MA share a common therapeutic target in the integrin family. Functional studies in Rax-CreER^T2^ transgenic mice confirmed that Itgb1 plays a critical role in maintaining the balance of hypothalamic GnRH–tanycyte unit during EA treatment.

**Conclusions:**

EA exerts therapeutic effects on PCOS by targeting hypothalamic GnRH–tanycyte unit, with Itgb1 identified as a key factor. MA primarily functions through neurotransmitter regulation. These findings highlight potential hypothalamic targets and provide new insights into the distinct mechanisms of EA and MA.

**Supplementary Information:**

The online version contains supplementary material available at 10.1186/s13020-025-01068-3.

## Introduction

Polycystic ovary syndrome (PCOS) is a prevalent endocrine disorder affecting 5–15% of reproductive-age women worldwide [1], leading to complications such as infertility, metabolic syndrome, and an increased risk of endometrial cancer [2–4]. Previous studies have proposed that hypothalamic dysfunction is a critical driver of PCOS [5, 6]. Specifically, dysregulation of the hypothalamic-pituitary-ovarian axis (HPOA) is a hallmark of PCOS and is prominently characterized by impaired control of gonadotropin-releasing hormone (GnRH) secretion [7, 8]. GnRH neurons establish extensive projection networks, with neuronal bodies predominantly located in the medial preoptic area (MPOA) of the hypothalamus, and axons extend to regions such as the arcuate nucleus (ARC) and the median eminence (ME) [9, 10].

Tanycytes are specialized glial cells that provide structural support and regulatory functions within the hypothalamus [11, 12]. The processes of tanycytes are closely intertwined with the axonal terminals of GnRH neurons [13], constituting the GnRH–tanycyte unit. Tanycytes exhibit remarkable plasticity, with processes capable of altering shape and number. Alterations in tanycytes influence the geometry of the extracellular space surrounding GnRH neurons, thereby affecting both the secretion rate of hormones and their diffusion into the bloodstream [14, 15]. Although previous studies have highlighted the crucial role of tanycytes in maintaining the homeostasis of GnRH release, the structural remodeling of tanycytes in the pathological state of PCOS and their regulatory impact on GnRH secretion remain incompletely understood.

Clinical studies have reported that acupuncture can improve menstrual regularity, reduce androgen levels, and increase fertility in women with PCOS [16, 17]. Electroacupuncture (EA) and manual acupuncture (MA) have been shown to regulate different neurotransmitters and hormone receptors in the hypothalamus, thereby improving the function of the HPOA [18, 19]. Research has shown that EA helps recover the estrous cycle in PCOS-like animals by decreasing androgen receptor (AR) expression in kisspeptin neurons within the hypothalamic ARC, contributing to decreased GnRH and LH levels [20]. Moreover, MA restored the estrous cycle and abnormal sex hormone levels in PCOS-like rats by reducing the mRNA expression levels of estrogen receptor β, progesterone receptor and kisspeptin receptor in the hypothalamus [21]. Acupuncture potentially influences neuroglial interactions and the morphological plasticity of glial cells involved in hormone regulation, which contributes to overall endocrine homeostasis [22]. However, the precise mechanisms and associated molecular pathways underlying the relationship between acupuncture and the GnRH–tanycyte unit remain poorly understood.

The aim of this study was to investigate the role of the GnRH–tanycyte unit in the treatment of PCOS through the application of EA and MA. By employing advanced tissue clearing techniques and transcriptome analysis, we focused on elucidating the 3D morphology and molecular characteristics of the GnRH–tanycyte unit. Our findings highlight the therapeutic potential and different targets of EA and MA in modulating the neuroendocrine network to alleviate PCOS-related disturbances.

## Materials and methods

### Animals

Three-week-old female C57BL/6 J mice and Wistar rats were purchased from Slack Laboratory Animal Center (Shanghai, China). The Rax-CreER^T2^; Ai14 mouse line was generously provided by Dr. Qingfeng Wu (Chinese Academy of Sciences, Beijing) [23]. The animals were housed under standard conditions (22–23 °C, 12–hour light/dark cycle) with unrestricted access to food and water. All animal experiments were conducted in compliance with the National Institutes of Health Guide for the Care and Use of Laboratory Animals (NIH Publication No. 8023, revised 1978) and approved by the Animal Care and Use Research Ethical Standards of Fudan University (No. 20210302-086, Shanghai, China).

### PCOS-like mouse model

At the age of three-week-old, the animals were randomly divided into four experimental groups (Sham, PCOS, PCOS + EA, and PCOS + MA) and subcutaneously implanted with eight-week continuous-release capsules containing 15 mg DHT (Cat#A8380, Sigma‒Aldrich), whereas the Sham group received empty capsules. Before implantation, the surgical site was shaved and disinfected, and capsules were inserted under sterile conditions. After three weeks of DHT modeling, the estrous cycles were determined by analyzing vaginal cells daily under a microscope: the proestrus stage (predominantly nucleated epithelial cells), the estrus stage (mainly cornified, anucleated cells), the metestrus stage (mixed leukocytes, cornified, and nucleated epithelial cells), and the diestrus stage (mostly leukocytes with few epithelial cells) [24].

### EA and MA treatments

Eight weeks after PCOS induction, acupuncture treatment was administered at two selected acupoints: “*Guilai*” (ST29) and “*Sanyinjiao*” (SP6). *Guilai* acupoints are typically located on the lower abdomen, approximately 3.5 mm lateral to the midline and 6.5 mm below the umbilicus*. Sanyinjiao* acupoints are located 5 mm above the medial malleolus of the hind limb [25]. Before needle insertion, the mice were mildly anesthetized with isoflurane for 2 minutes (min). Acupuncture needles (Φ 0.22 × 13 mm) were swiftly inserted 2–3 mm into the acupoints. Throughout the treatment, the mice remained conscious and were gently suspended in a fabric harness above the table. Both the EA and MA treatments were administered five days per week for a total duration of four weeks [26, 27].

EA: The needles were connected to a HANS-200 electroacupuncture device (Nanjing Jisheng Medical Technology Co., Ltd., Nanjing, China), and a stimulus was applied at 2 Hz with burst pulses. The intensity was adjusted to induce regular leg muscle contractions (0.2–0.5 mA). Each treatment session involved 30 min of electrical stimulation.

MA: Needles were inserted into the designated acupoints and manually manipulated with a combination of techniques, including lifting, thrusting, twisting, and rotating. Each manipulation session lasted for 1 min, followed by a 5-min interval. The needles were twisted at a standardized frequency of 60 twists per minute. The procedure was performed by the same researcher to equalize the stimulation of the acupoints. The manually stimulation was carefully maintained for 30 min, ensuring consistency and even pressure throughout the session.

### Hematoxylin and eosin (HE) staining

Ovarian tissues were collected and fixed in paraformaldehyde (PFA) for 24 h. The samples were then dehydrated through a graded series of ethanol, cleared in xylene, and embedded in paraffin. Serial 5 µm thick sections were obtained using a microtome and mounted on glass slides. For HE staining, the sections were deparaffinized, rehydrated, and stained with hematoxylin for 5 min, followed by incubation with eosin for 2 min. After dehydration and clearing, the stained sections were coverslipped with resinous mounting medium. Ovarian morphology, including follicular development stages, was then examined under a light microscope.

### Immunofluorescence (IF) staining

IF staining of mouse hypothalamic tissue began with tissue fixation with 4% PFA overnight, followed by dehydration in graded sucrose solutions. Brain sections (30 µm) were prepared with a microtome, permeabilized with 0.2% Triton X-100, and blocked with 5% normal serum. The sections were incubated overnight at 4 °C with the following primary antibodies: rabbit anti-GnRH (1:2000 dilution, Cat# G8294, Sigma‒Aldrich), chicken anti-vimentin (1:1000 dilution, Cat# AB5733, Millipore), and rabbit anti-integrin beta 1 (1:500 dilution, Cat# 12594-1-AP, Proteintech). Fluorophore-conjugated secondary antibodies were applied for 2 h at room temperature. After DAPI staining, the sections were mounted and imaged using confocal microscopy (NCF950, Ningbo Yongxin Optics Co., Ltd.). High-resolution confocal imaging was performed using a Nikon spatial array confocal system (NSPARC) to analyze ultrastructure. Imaris software (v9.8, Andor Technology, Britain), ImageJ-Pro Plus 6.0 (Media Cybernetics), and NIS-Elements Viewer 5.21 (Nikon Corporation) were used for fluorescence intensity quantification.

### Immunolabeling-enabled 3D imaging of solvent-cleared organs (iDISCO)

The iDISCO protocol for processing hypothalamic tissue involves several steps, including dehydration, antibody incubation, and refractive index matching [28]. We selected rat brain samples over mouse samples for transparency studies because of the larger size of the rat brain, which provides improved anatomical resolution and greater detail of GnRH neurons. Rat hypothalamus were first dehydrated with a graded methanol series and then gradually transitioned to pure methanol. The samples were then incubated with GnRH antibody (1:100 dilution, Cat# G8294, Sigma‒Aldrich) for 5 days at 37 °C. After incubation, the excess antibodies were removed by washing with PTWH (PBS + 0.1% Tween 20). The tissues were then optically cleared by refractive index matching using Dibenzyl Ether (DBE, Cat# 33630, Sigma‒Aldrich). The cleared samples were imaged using a light sheet microscope (LS18, Nuohai Life Science), which enabled 3D visualization of GnRH-positive neurons within the hypothalamus. The ovaries were dehydrated and stained with tyrosine hydroxylase (TH, 1:50 dilution, Cat#ab112, Abcam) and DAPI (1:100 dilution, Cat#62248, Thermo Fisher Scientific), followed by tissue transparency, with the processing time reduced by half.

The “*Surface*” algorithm in Imaris software was utilized for 3D visualization and quantification. The process begins by selecting the “*Surface*” tool from the toolbar and configuring parameters such as intensity thresholding and background subtraction. The Imaris software then generates a 3D reconstruction of detected objects. Additional refinements, including smoothing, segmentation, and threshold adjustments, can be applied to improve surface detection.

### Enzyme-linked immunosorbent assays (ELISA)

Blood samples were collected by cardiac puncture and centrifuged at 3000 rpm for 15 min to separate the serum. ELISAs were performed to measure the serum levels of luteinizing hormone (LH; BPE20343), follicle-stimulating hormone (FSH; BPE20419), dihydrotestosterone (DHT; BPE20577), testosterone (T; BPE20375), estradiol (E2; BPE20381), free testosterone (fT; BPE20374), and sex hormone-binding globulin (SHBG; BPE21020; Lengton Biological Technology). The ELISA kits demonstrated an interassay variability of 12% and an intra-assay variability of 10%, ensuring consistent and reliable measurements of sex hormones across experiments. Each serum sample was analyzed in duplicate, with standards prepared in serial dilutions. The absorbance (OD) was measured using a spectrophotometer at 450 nm. The standard curve regression equation was calculated using ELISAcalc software, which applies a logistic four-parameter model for curve fitting based on concentration and OD values.

### Polymerase chain reaction (PCR)

Total RNA was extracted from mouse hypothalamus using TRIzol reagent (Invitrogen, Thermo Fisher Scientific), followed by phase separation with chloroform and isopropanol precipitation. The RNA pellet was washed with 75% ethanol, air-dried, and resuspended in RNase-free water. The RNA concentration and purity were assessed using a NanoDrop spectrophotometer. Subsequently, 1 µg of RNA was reverse-transcribed into cDNA (Cat# RR036A, TaKaRa). Quantitative PCR (qPCR) was performed using TB Green^®^ *Premix Ex Taq*^™^ (Cat# RR420A, TaKaRa) and gene-specific primers (Supplementary Table 1 for primer sequences of related genes), with GAPDH serving as the internal control. Careful handling and the use of RNase-free materials were maintained throughout the procedure to ensure sample integrity and prevent contamination.

### Western blot (WB)

Proteins from the mouse hypothalamus were extracted with RIPA buffer (Cat# P0013B, Beyotime) supplemented with protease and phosphatase inhibitors. The lysates were incubated on ice for 30 min, followed by centrifugation at 12,000 × *g* for 15 min to collect the supernatant. Equal amounts of denatured protein samples were resolved by SDS‒PAGE and transferred to PVDF membranes. After blocking in 5% nonfat milk, the membranes were incubated overnight at 4 °C with primary antibodies against AR (1:1000 dilution, Cat# ab133273, Abcam), Itgb1 (1:1000 dilution, Cat# 12594-1-AP, Proteintech), focal adhesion kinase (FAK, phospho Y397) (1:500 dilution, Cat# ab81298, Abcam), FAK (1:500 dilution, Cat# ab40794, Abcam), TGF beta receptor I (phospho S165) (1:1000 dilution, Cat# ab112095, Abcam), TGF beta receptor I (1:1000 dilution, Cat# ab121024, Abcam), Smad2 (phospho S467) (1:500 dilution, Cat# ab280888, Abcam), and Smad2 (1:500 dilution, Cat# ab33875, Abcam). After washing, the membranes were incubated with HRP-conjugated secondary antibodies for 2 h. The protein bands were visualized using enhanced chemiluminescence (ECL, WBKLS0500, Millipore) and imaged using an Image Quant LAS4000 mini-gel imaging system (GE Healthcare Life Sciences). All specific protein band densities were normalized to those of GAPDH or the corresponding total protein as the loading control and analyzed using Image-Pro Plus 6.0.

### Transcriptome analysis

Transcriptome was conducted by OE Biotech Co., Ltd. (Shanghai, China). Total RNA was extracted from hypothalamic samples from the Sham, PCOS, PCOS + EA, and PCOS + MA groups using TRIzol following the manufacturer’s protocol. The RNA purity and concentration were evaluated using a Nanodrop2000 (Thermo Scientific, USA). Libraries were constructed using the VAHTS Universal V6 RNA-seq Library Prep Kit and sequenced on an Illumina NovaSeq 6000 platform to generate 150 bp paired-end reads. Clean reads were obtained by filtering raw reads with fastp [29] and then aligned to the mouse reference genome using HISAT2 [30]. Gene expression was quantified as FPKM values, with read counts generated using HTSeq-count [31]. Differential gene expression analysis was conducted using DESeq2 [32]. P < 0.05 and |log FC|> 0.585 were considered indicative of significant differential expression.

### Specific knockdown of* Itgb1* in tanycytes

Rax-CreER^T2^ transgenic mice were subjected to PCOS modeling at three weeks of age. Six weeks after PCOS modeling, the mice underwent stereotactic injection of DIO-EGFP shRNA (*Itgb1*) virus (serotype 1:2 chimeric; titer = 5 × 10^10^, BrainVTA) to achieve tanycyte-specific gene silencing. Stereotactic injection was precisely guided to target the lateral ventricle (2 μL; at 0.2 μL/min; anteroposterior, − 0.3 mm; midline, − 1 mm; dorsoventral, − 2.5 mm), ensuring accurate delivery of the viral construct. The inclusion of the EGFP reporter enabled visual confirmation of successful viral transduction and tanycyte targeting. Tamoxifen (100 mg/kg, HY-13757A, MCE, China) was administered intraperitoneally for five consecutive days to induce Cre recombinase expression specifically in tanycytes.

### Statistical analysis

All the statistical analyses were conducted in GraphPad Prism (v9.0). Normality was assessed using the Shapiro‒Wilk test and Q‒Q plots, whereas homogeneity of variance was evaluated using the Brown‒Forsythe test to ensure the appropriateness of the statistical methods. The data are expressed as the mean ± standard error of the mean (SEM). For comparisons between two groups, an independent samples *t- *test was performed. For multiple group comparisons, one-way or two-way ANOVA was used. Statistical significance was set at *p* < 0.05.

## Results

### EA and MA alleviated reproductive and endocrine disorders in PCOS-like mice

**Fig.1:**
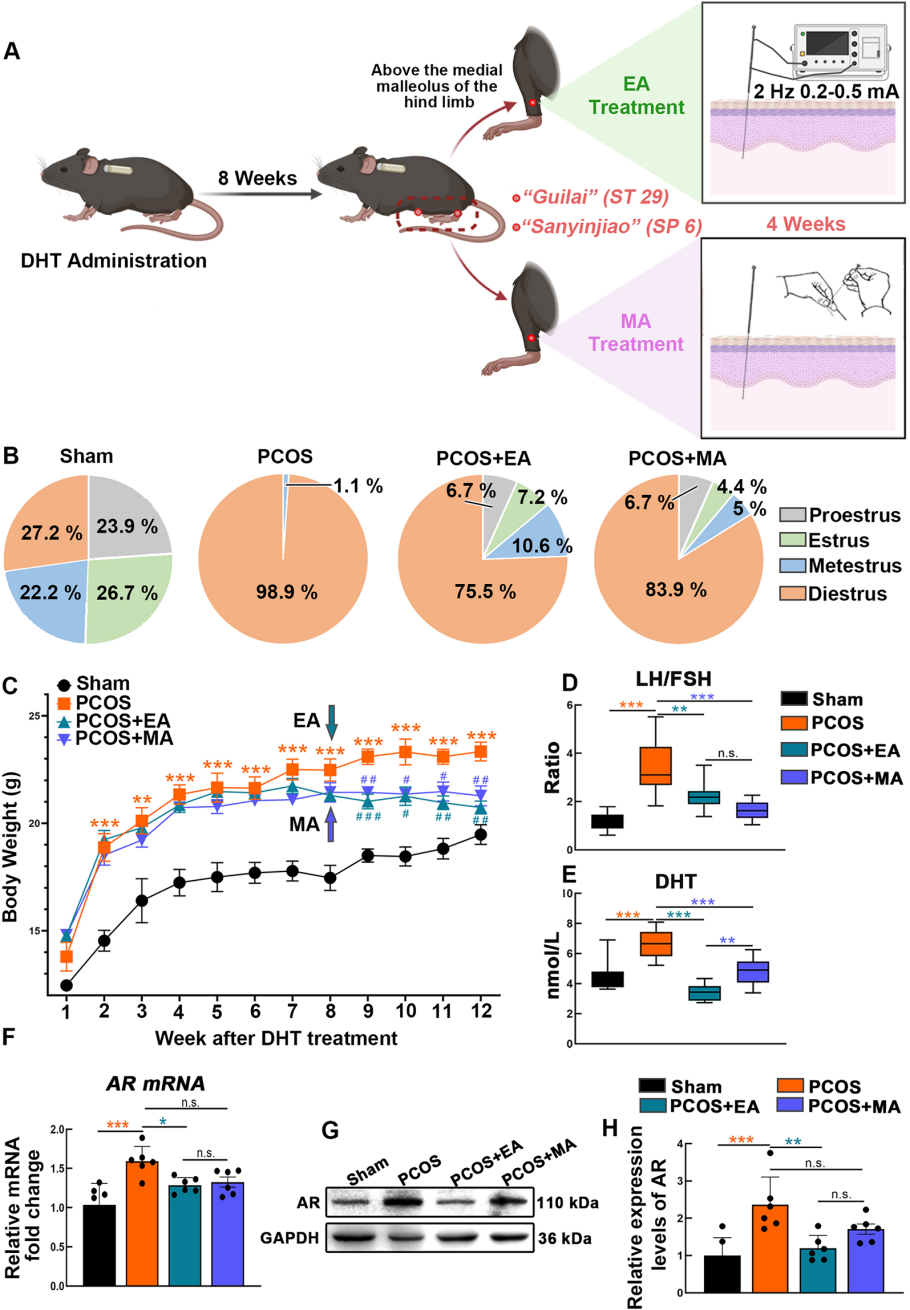
Acupuncture improves reproductive endocrine dysfunction in PCOS. **A** Schematic representation of the experimental: Mice were administered DHT for 8 weeks to establish PCOS-like model, followed by 4 weeks of EA or MA treatment at two acupoints (*Guilai*, ST 29; *Sanyinjiao*, SP 6). **B** Quantitative analysis of the cycle stages. Pie charts depicted the percentage of days spent in the different stages of the estrus cycle in the Sham, PCOS, PCOS + EA, and PCOS + MA groups (*n* = 5). **C** Body weight curves over 12 weeks showing changes in different groups (*n* = 5, Statistical analysis: One-way ANOVA, Dunnett’s post hoc test. **p < 0.01, ***p < 0.001. * *vs.* Sham group; #*p* < 0.05, # #*p* < 0.01, # # #*p* < 0.01, # *vs.* PCOS group). **D**, **E** Box plot of LH/FSH ratios and DHT levels (nmol/L) among the four groups (*n* = 10, Statistical analysis: One-way ANOVA, **(D)** F_(3,36)_ = 19.99, p < 0.0001; **(E)** F_(3,36)_ = 22.47, p < 0.0001; Tukey’s post hoc test. **p < 0.01, ***p < 0.001). **F** Relative *AR* mRNA expression levels measured by RT-PCR in the hypothalamus (*n* = 6, Statistical analysis: One-way ANOVA, F_(3,20)_ = 8.680, p = 0.0007; Dunnett’s post hoc test. *p < 0.05, ***p < 0.001). **G**, **H** Representative protein bands and quantification of AR levels in the hypothalamus with GAPDH as a loading control (*n* = 6, Statistical analysis: One-way ANOVA, F_(3,20)_ = 8.827, p = 0.0006; Tukey’s post hoc test. **p < 0.01, ***p < 0.001)

A schematic diagram depicting PCOS modeling, along with the treatment protocols for EA and MA, is shown in Fig. 1A. The estrous cycle serves as an indicator for assessing reproductive function. PCOS-like mice predominantly remained in the diestrus phase (D, the nonreceptive stage). EA and MA treatments restored a regular estrous cycle, with a higher proportion of mice in the estrus phase (E) observed in the PCOS + EA group than in the PCOS + MA group (Fig. 1B and Fig. S1A). HE staining revealed arrested folliculogenesis with antral follicles in PCOS ovaries. The PCOS + EA group presented 2–3 mature follicles, whereas the PCOS + MA group presented less improvement, highlighting the superior regulatory effect of EA on estrous cycle recovery and ovarian development (Fig. S1B).

With continued weight monitoring, the PCOS-like mice presented a significant increase in body weight, whereas both the EA and MA treatments resulted in lower gains in body weight (Fig. 1C). The LH/FSH ratios were significantly decreased in both the EA- and MA-treated groups (Fig. 1D). In addition, the levels of DHT (Fig. 1E), total testosterone (Fig. S2 A), and free testosterone (Fig. S2 B) were notably lower in the PCOS + EA group, although these reductions were less pronounced in the PCOS + MA group. Serum estradiol (Fig. S2 C) and SHBG levels (Fig. S2 D) were increased in both acupuncture groups. The increased reproductive organ weights (Fig. S3) in the EA and MA groups reflected improved ovarian function, suggesting normalization of the hormonal environment and increased reproductive capacity.

Furthermore, we assessed the mRNA and protein expression levels of AR in the hypothalamus to evaluate peripheral androgen signaling invasion. The results revealed a significant increase in hypothalamic AR expression in PCOS-like mice, whereas EA dramatically reduced AR levels (Fig. 1F–H). These findings highlight the ability of EA to target the hypothalamus to relieve endocrine dysfunction related to excess androgens in individuals with PCOS, whereas MA has minimal effects on AR regulation.

### EA, but not MA, restored the expanded morphology and distribution of GnRH neurons in the hypothalamus

**Fig. 2:**
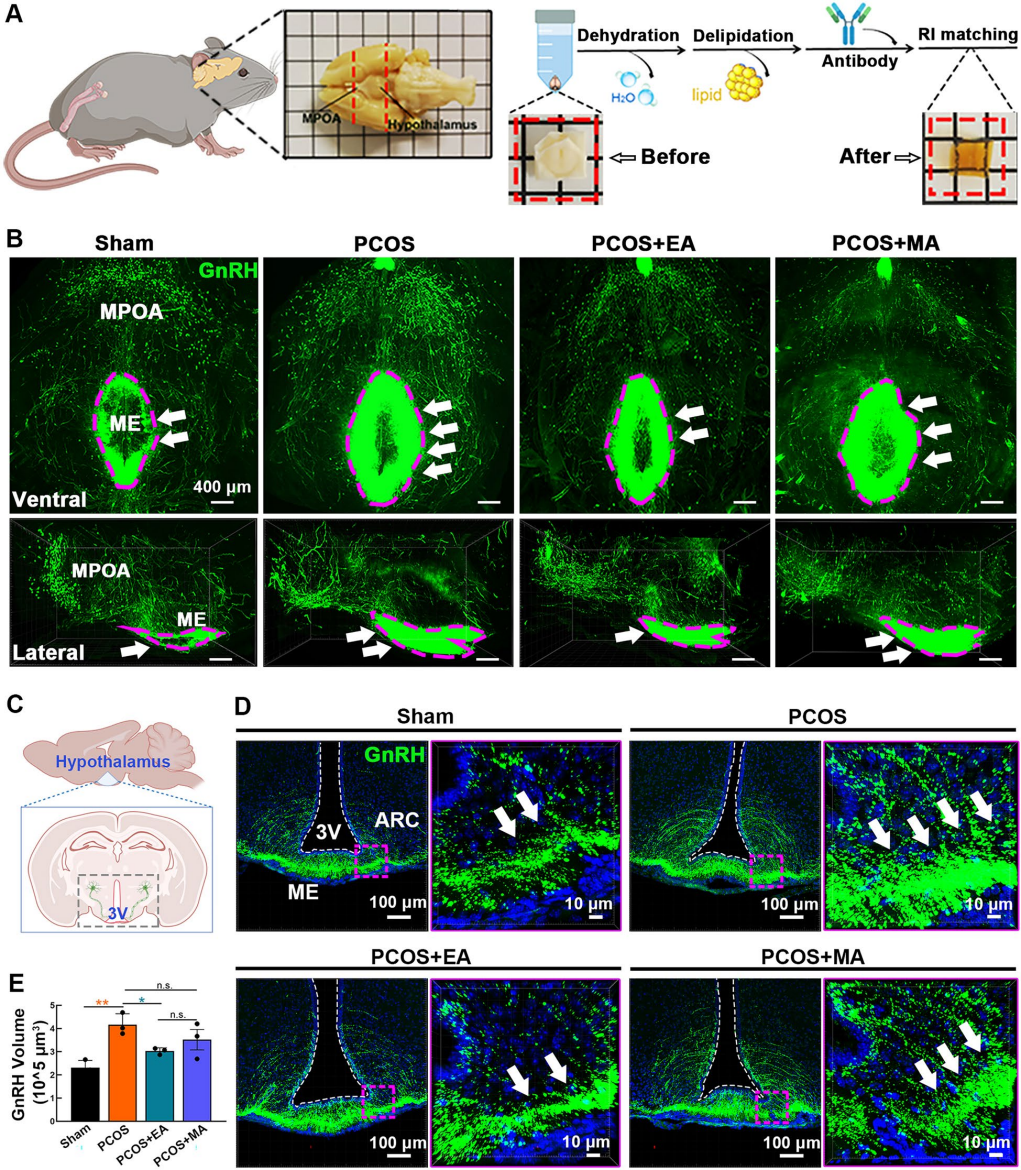
Three-dimensional visualization of hypothalamic GnRH neurons and acupuncture-induced changes. **A** Workflow diagram illustrating the iDISCO tissue clearing protocol used for visualizing hypothalamic GnRH neurons. **B** Ventral and lateral views of hypothalamic GnRH neurons in the Sham, PCOS, PCOS + EA, and PCOS + MA groups. GnRH-positive projections extend from the MPOA to the ARC and ME, with the purple dashed line encircling the positive signal clusters formed by GnRH neuron axon terminals. The arrows indicate changes in the volume of these signal clusters. **C** Schematic diagram highlighting the anatomical region of the hypothalamus analyzed, including the ARC, ME, and 3 V. **D** Representative high-resolution images of GnRH-positive axons (green) in the ME and ARC across Sham, PCOS, PCOS + EA, and PCOS + MA groups. **E** Quantitative analysis of GnRH-positive axon volume (10^5 µm^3^). (*n* = 3, Statistical analysis: One-way ANOVA, F_(3,8)_ = 7.932, p = 0.0088; Tukey’s post hoc test. *p < 0.05, **p < 0.01)

The iDISCO technique was used to make hypothalamic samples transparent after gradient dehydration, antibody incubation, and refractive index matching with DBE (Fig. 2A). In the Sham group, GnRH neuron cell bodies were located primarily in the MPOA, with axons projecting to the ARC and ME regions, thus forming small and evenly distributed signal clusters. In contrast, PCOS-like mice presented thicker and denser axonal projections that terminated near the third ventricle (3V). These abnormalities were alleviated in the PCOS + EA group, in which the size of the signal clusters was reduced. However, the PCOS + MA group still presented larger signal clusters and irregular axonal structures, closely resembling those observed in the PCOS model (Fig. 2B). We utilized a high-resolution confocal microscope to visualize the distribution and aggregation of GnRH axon terminals in the hypothalamic ME and ARC. PCOS-like mice presented a significant increase in GnRH-positive axonal projections (green). EA treatment significantly reduced the abnormal aggregation. In contrast, MA had less pronounced effects (Fig. 2C, D). Quantitative analysis of GnRH volume further confirmed that EA markedly decreased GnRH aggregation compared with that in the PCOS groups (Fig. 2E). These results suggest that EA more effectively prevents excessive accumulation of GnRH neuron axons in the basal hypothalamus of PCOS-like animals, thereby alleviating reproductive endocrine disorders.

### Comparison of transcriptomic changes in the hypothalamus induced by EA and MA

**Fig. 3:**
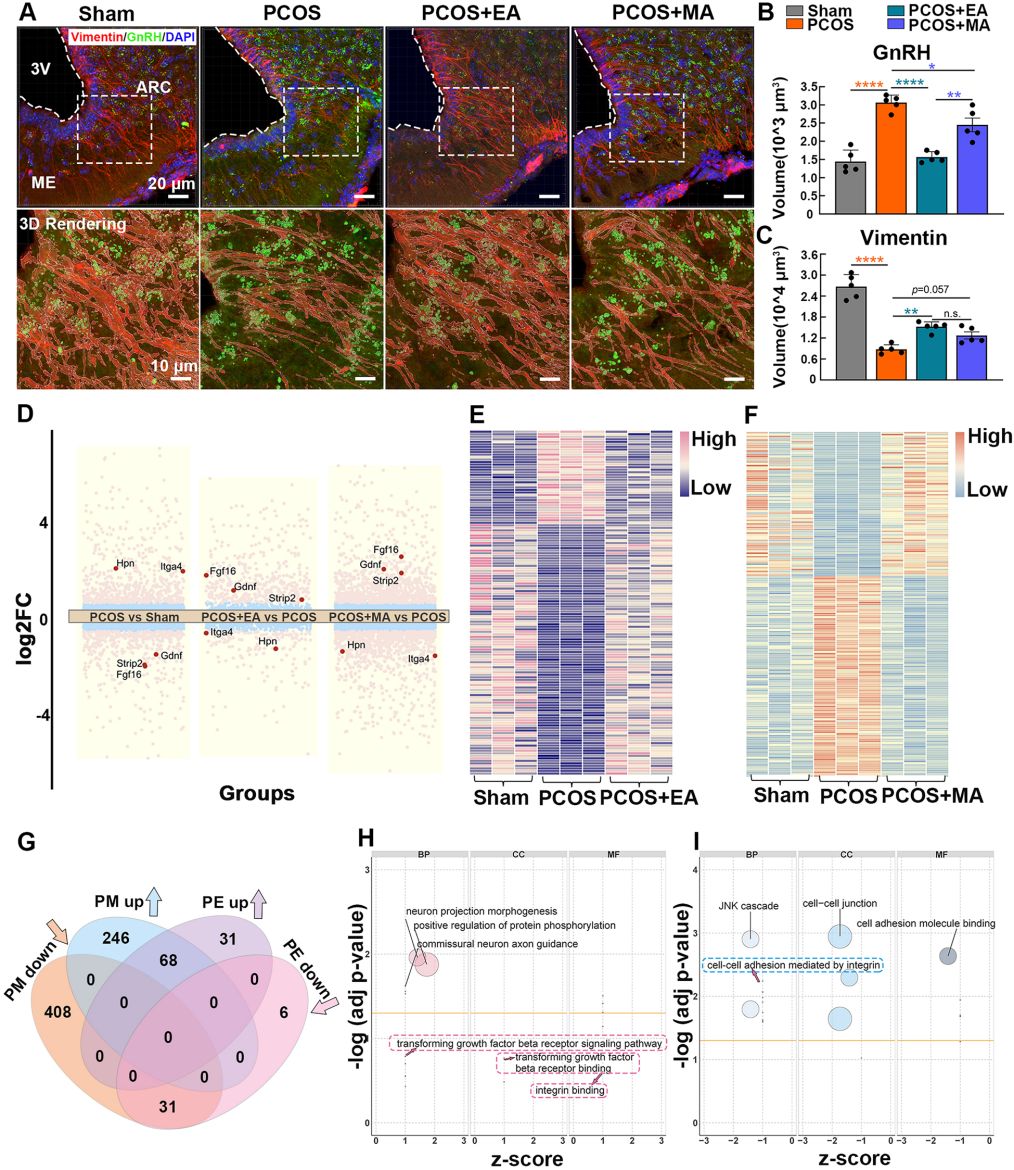
Transcriptome sequencing analysis of DEGs and functional implications. **A** IF staining and 3D reconstruction of GnRH and Vimentin in the hypothalamic ARC and ME regions across different groups, with quantification of GnRH-positive axonal projections (**B**) and Vimentin-positive tanycyte processes (**C**) (*n* = 5, Statistical analysis: One-way ANOVA, **(B)** F_(3,16)_ = 34.38, p < 0.0001; **(C)** F_(3,16)_ = 58.23, P < 0.0001; Tukey’s post hoc test. *p < 0.05, **p < 0.01, ****p < 0.0001). **D** Volcano plots showing key DEGs across PCOS *vs.* Sham, PCOS + EA *vs.* PCOS, and PCOS + MA *vs*. PCOS groups. **E**, **F** Heatmaps displaying gene expression profiles in Sham, PCOS, PCOS + EA, and PCOS + MA groups. **G** Venn diagram illustrating the overlap of DEGs between PCOS + EA and PCOS + MA groups, indicating both unique and shared gene expression changes. GO enrichment analysis highlighting functions associated with upregulated (**H**) and downregulated (**I**) genes in both EA and MA treatments

Given the potential role of tanycytes in regulating GnRH neurons, we investigated the morphological and functional alterations in the GnRH–tanycyte unit in the pathological state of PCOS, as well as the regulatory effects of EA and MA on this unit. Figure 3A illustrates the morphological and distributional changes in the GnRH–tanycyte unit within the ARC and ME regions. The PCOS + EA groups presented reduced GnRH signals with denser tanycyte processes. Quantification analysis confirmed elevated GnRH-positive volumes in PCOS-like mice, with EA treatment significantly reducing GnRH signals and enhancing tanycyte morphology. The changes observed in the MA group were smaller and not statistically significant (Fig. 3B, C).

To further elucidate the molecular targets of acupuncture regulation of GnRH–tanycyte unit, we conducted transcriptome sequencing of the hypothalamus. A volcano plot was constructed to illustrate the changes in gene expression (Fig. 3D), highlighting key genes with significant differential expression across the PCOS *vs.* Sham (up 629, down 482), PCOS + EA *vs.* PCOS (up 420, down 159), and PCOS + MA *vs.* PCOS groups (up 944, down 972). Principal component analysis (PCA, Fig. S4) and heatmaps (Fig. 3E, F) revealed distinct clustering of gene expression profiles after the EA and MA treatments. Venn diagrams displayed the overlap of differentially expressed genes (DEGs) among the comparisons, with the EA and MA treatments sharing a substantial number of up-regulated and down-regulated genes with the PCOS *vs.* Sham comparison (Fig. S5).

GO and KEGG functional enrichment analyses of DEGs in the EA and MA groups revealed distinct targets within the hypothalamus. EA treatment primarily targeted pathways essential for maintaining neuroglial plasticity, including neuron projection morphogenesis, cell-cell junctions, astrocyte development, and structural integrity. Furthermore, EA modulated key signaling pathways, such as the PI3K-Akt, TGF-β, and Hippo pathways, which regulate cellular architecture and organ morphology [33], further emphasizing its role in promoting neuroglial plasticity (Fig. S6). In contrast, the MA group was more prominently enriched in pathways associated with dopamine receptor activity, neuronal cell bodies, the synaptic vesicle membrane, and GABAergic synapses, highlighting its specific regulatory effects on neurotransmitter release and neuropeptide regulation (Fig. S7).

Additionally, the EA and MA groups shared common molecular targets. Venn diagram revealed the overlap of DEGs between the PCOS + EA *vs.* PCOS (PE up and PE down) and PCOS + MA *vs.* PCOS (PM up and PM down) groups, highlighting both common and unique gene profile changes between treatments (Fig. 3G). We further conducted functional enrichment analysis on genes commonly up- and down-regulated in both the EA and MA groups compared with the PCOS group. Figure 3H highlights the upregulated functions, including neuron projection morphogenesis, astrocyte activation, the TGF-β receptor, and integrin binding. Figure 3I shows processes such as hormone biosynthesis, the regulation of neuroinflammatory response, and integrin-mediated cell-cell adhesion, indicating that EA and MA modulate shared pathways involved in integrin family and cellular structure regulation.

### EA restored hypothalamic Itgb1 signaling in PCOS-like mice

**Fig. 4:**
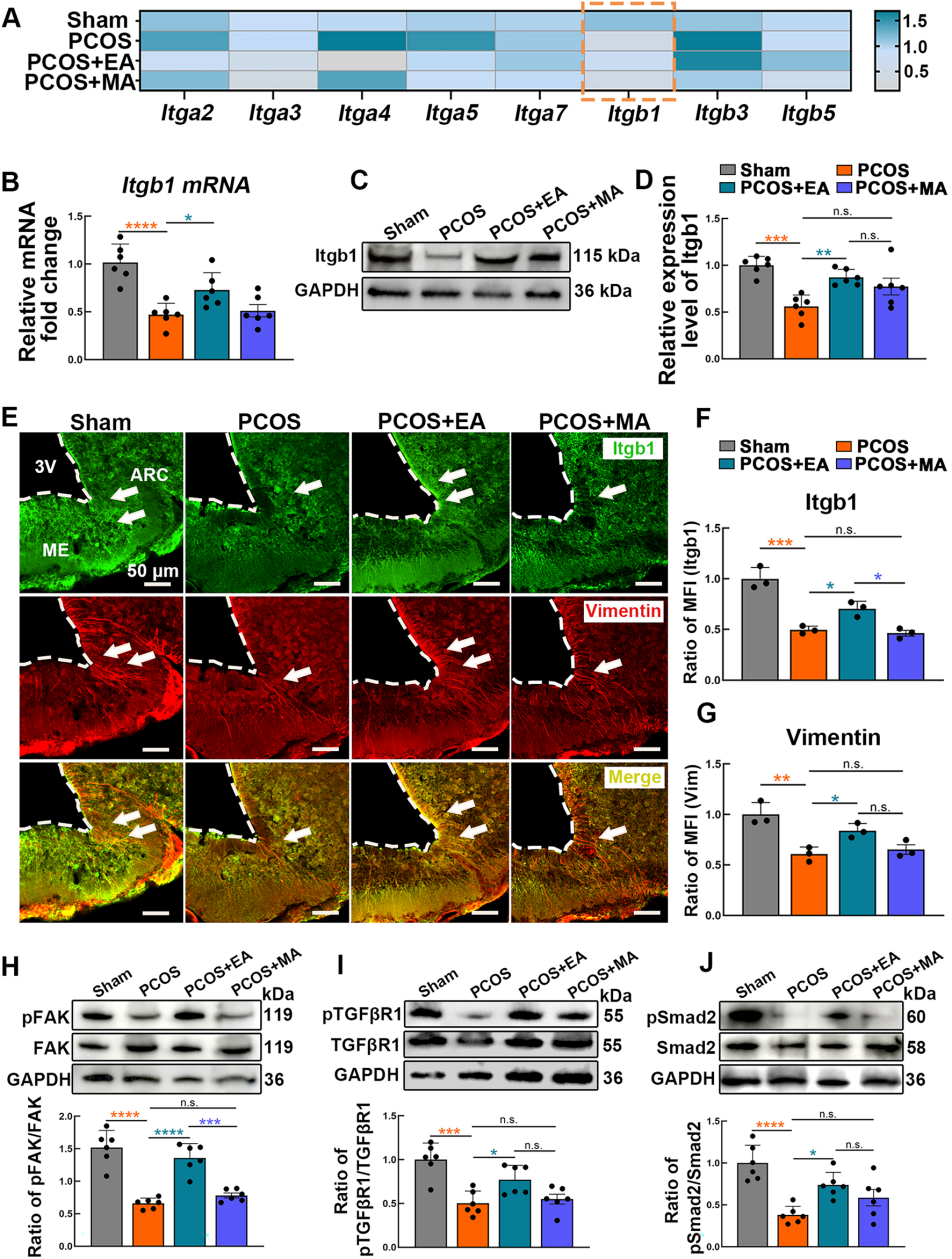
EA modulates Itgb1 expression in the hypothalamus and activates the associated signaling pathway. **A** Heatmap depicting mRNA expression levels of integrin subunits (*Itga2, Itga3, Itga4, Itga5, Itga7, Itgb1, Itgb3, Itgb5*) across the Sham, PCOS, PCOS + EA, and PCOS + MA groups. **B** Quantification of *Itgb1* mRNA levels by RT-PCR, showing relative fold changes (*n* = 6, Statistical analysis: One-way ANOVA, F_(3,20)_ = 13.66, p < 0.0001; Tukey’s post hoc test. *p < 0.05, ****p < 0.0001). **C**, **D** Representative bands and grayscale values of Itgb1 protein expression in the hypothalamus (*n* = 6, Statistical analysis: One-way ANOVA, F_(3,20)_ = 10.49, p = 0.0002; Tukey’s post hoc test. **p < 0.01, ***p < 0.001). **E** IF images of Itgb1 (green) and vimentin (red) in the hypothalamus, focusing on the ARC and ME regions. The dashed lines delineate tissue boundaries, and arrows indicate key regions of interest. **F** Quantification of mean fluorescence intensity (MFI) of Itgb1 across different groups (*n* = 3, Statistical analysis: One-way ANOVA, F_(3,8)_ = 33.49, p < 0.0001; Tukey’s post hoc test. *p < 0.05, ***p < 0.001). **G** Quantification of vimentin MFI as a marker of tanycyte morphology (*n* = 3, Statistical analysis: One-way ANOVA, F_(3,8)_ = 12.58, P = 0.0021; Tukey’s post hoc test. *p < 0.05, **p < 0.01). **H**–**J** Western blot analysis of phosphorylated and total levels of key proteins in the FAK/TGF-βR1/Smad2 signaling pathway: FAK (**H**), TGF-βR1 (**I**), and Smad2 (**J**). Densitometric quantification of phosphorylation ratios is shown below each blot (*n* = 6, Statistical analysis: One-way ANOVA, (**H**) F_(3,20)_ = 32.25, P < 0.0001; (**I**) F_(3,20)_ = 12.29, p < 0.0001; (**J**) F_(3,20)_ = 12.05, p < 0.0001;Tukey’s post hoc test. *p < 0.05, ***p < 0.001, ****p < 0.0001)

Building on the transcriptome sequencing results, which highlighted the potential roles of the integrin family, we further explored the role of integrins in regulating the GnRH–tanycyte unit. We first analyzed the transcription levels of integrin family genes potentially associated with PCOS (Fig. 4A). The expression of Itgb1, a key target, was significantly reduced at both the mRNA and protein levels in PCOS-like mice. Notably, EA treatment significantly upregulated Itgb1 expression, whereas MA treatment resulted in a moderate trend (Fig. 4B–D). IF staining was used to assess the spatial distribution and cellular localization of Itgb1 within the hypothalamus (Fig. 4E). In Sham mice, Itgb1 was broadly expressed in the ARC and ME regions, whereas PCOS-like mice presented a notable reduction in Itgb1-positive signals alongside fewer tanycyte processes. Intriguingly, the PCOS + EA group presented increased Itgb1 signals and denser tanycyte processes, highlighting the effectiveness of EA in restoring Itgb1 levels under PCOS conditions (Fig. 4E–G).

The FAK/TGF-βR1/Smad2 signaling pathway, which is mediated by Itgb1, plays crucial roles in cell survival and morphological adjustment [34, 35]. Transcriptome sequencing revealed significant involvement of the TGF-β signaling pathway in the therapeutic effects of acupuncture treatment (Figs. 3H, S6, and S7). Consequently, WB was performed to assess changes in the activity of the Itgb1-mediated FAK/TGF-βR1/Smad2 pathway following EA and MA treatments. The phosphorylation levels of key proteins, including pFAK, pTGF-βR1, and pSmad2, were significantly reduced in PCOS-like mice but were reactivated by EA, with MA showing a milder effect (Fig. 4H–J). These results indicate that EA effectively restores the expression of Itgb1 and reactivates the FAK/TGF-βR1/Smad2 signaling pathway, alleviating morphological and functional abnormalities of the GnRH–tanycyte unit in PCOS-like mice.

### Specific knockdown of *Itgb1* in tanycytes eliminated the therapeutic effect of EA

**Fig. 5:**
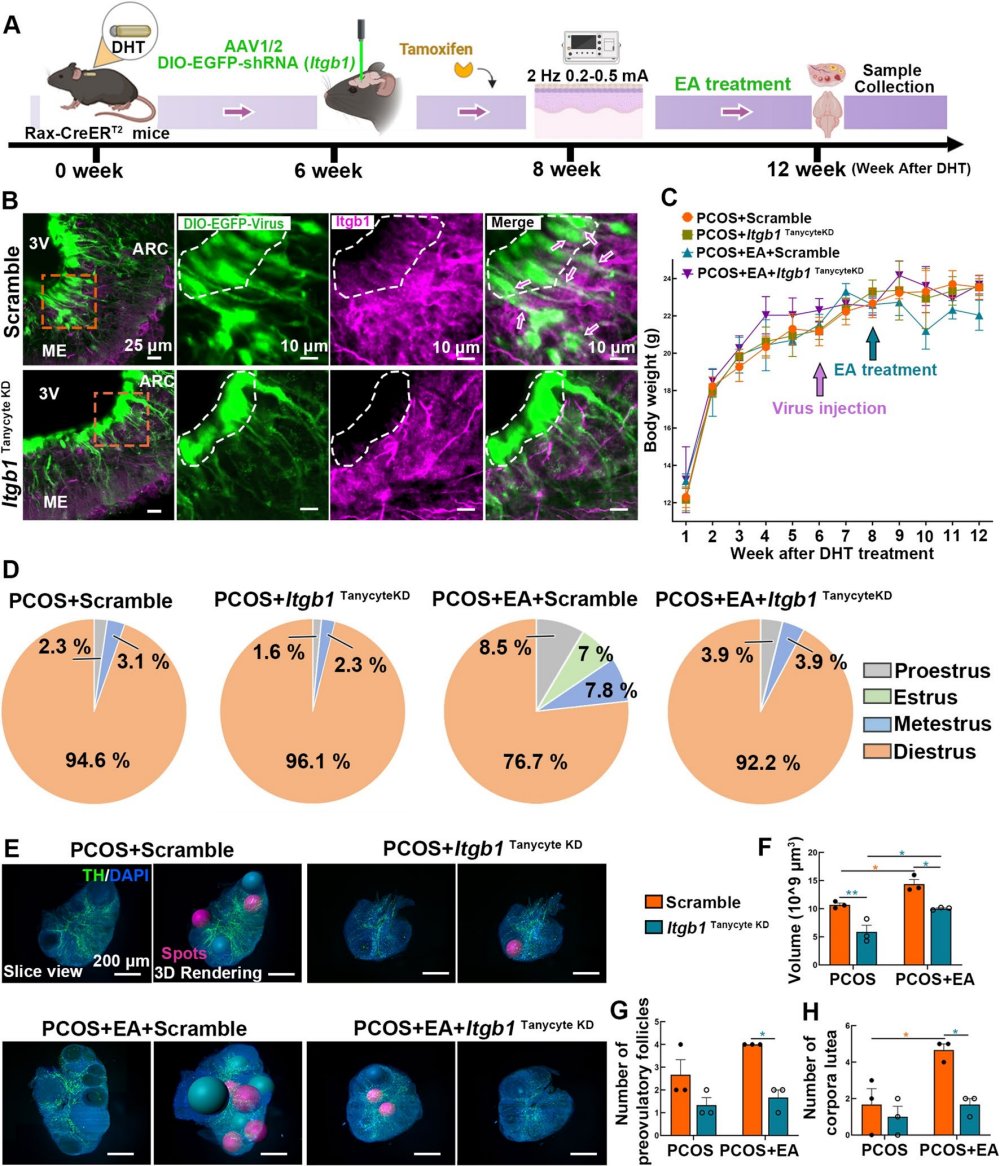
Selective *Itgb1* knockdown in tanycytes negates the therapeutic effects of EA. **A** The schematic diagram illustrates the experimental timeline and interventions applied to the PCOS model. AAV1/2 carrying DIO-EGFP-shRNA targeting *Itgb1* was delivered to the lateral ventricle. EA treatment was administered at a frequency of 2 Hz, 0.2–0.5 mA for four weeks. **B** Representative confocal images show Itgb1 expression (magenta) in the ARC and ME, alongside DIO-EGFP virus (green) in tanycytes of PCOS-like mice with either scrambled or *Itgb1* knockdown (Tanycyte KD). **C** The line graph showed body weight measurements over the 12-week period post-DHT treatment (*n* = 3). **D** Pie chart show estrous cycle phases monitored over time for each experimental group. **E** Representative three-dimensional images of ovaries stained by TH (green) and DAPI (blue) antibodies. Preovulatory follicles (magenta) and corpora lutea (cyan) were identified by the *Spots* algorithm. **F**–**H** Quantification of ovarian volume, number of preovulatory follicles and corpus lutea in each group (*n* = 3, Statistical analysis: Two-way ANOVA, Tukey’s post hoc test. *p < 0.05, **p < 0.01)

Given that EA had a stronger influence on Itgb1 and tanycytes, we prioritized EA for further investigation. We employed Rax-CreER^T2^ transgenic mice combined with a DIO-shRNA (*Itgb1*) virus to specifically knock down *Itgb1* in tanycytes. Six weeks after DHT administration, the shRNA (*Itgb1*)-producing virus was injected, followed by tamoxifen injection to activate Cre recombinase (Fig. 5A). IF staining confirmed the extensive infection of tanycytes and their processes by the DIO-EGFP virus. Mice injected with the scramble virus presented relatively high Itgb1 expression, with widespread colabeling with tanycytes. In contrast, tanycytic Itgb1 expression was reduced following virus injection, confirming the specificity of the virus in targeting and knocking down *Itgb1* in tanycytes (Fig. 5B).

Similarly, EA treatment reduced the body weight of PCOS-like mice injected with the scramble virus, demonstrating the effectiveness of EA. However, in mice administered with the *Itgb1*-specific knockdown virus, EA failed to reduce body weight (Fig. 5C). Estrous cycle monitoring revealed that EA restored the normal estrous cycle in scramble virus-injected mice but not in those with *Itgb1* knockdown, underscoring the fundamental role of Itgb1 in EA-induced cycle regulation (Fig. 5D). Three-dimensional imaging technology based on tissue transparency was employed to assess ovarian volume and follicle count in each group, providing an evaluation of ovarian ovulation function (Fig. 5E). The PCOS + EA + Scramble group presented increased ovarian volume, along with higher counts of preovulatory follicles and corpus luteum, compared to the PCOS + Scramble group. However, following injection of the *Itgb1 -*knockdown virus, the regulatory effects of EA were diminished, as evidenced by a reduction in ovarian volume and a decrease in the number of follicles across all stages (Fig. 5F–H).

### Itgb1 mediates EA regulation on GnRH–tanycyte unit via the FAK/TGF-βR1/Smad2 pathway

**Fig. 6:**
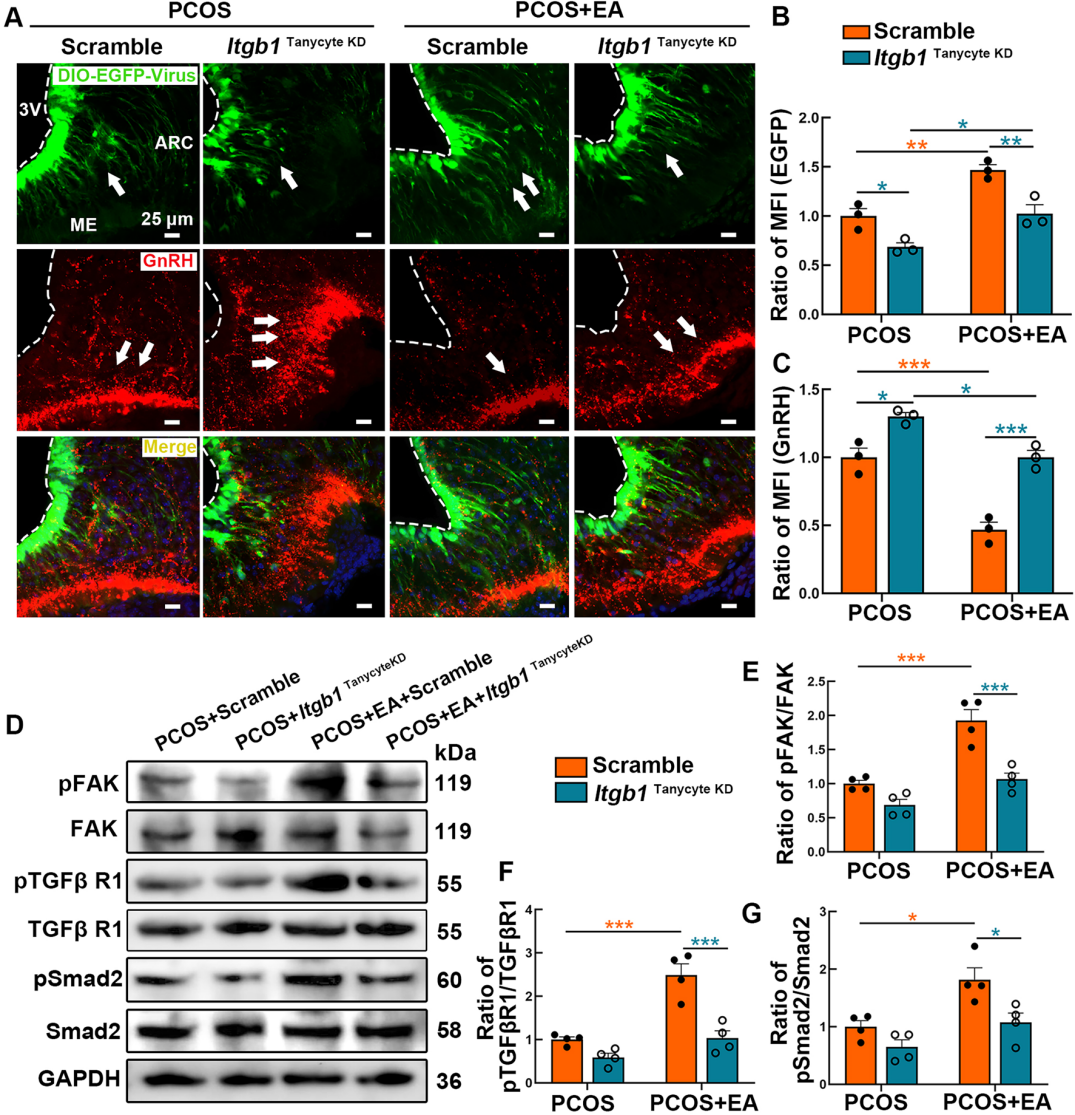
Itgb1 mediates the effects of EA on tanycyte function and GnRH axon dynamics. **A**. Confocal images illustrate the distribution of the DIO-EGFP virus (green) in the ME, accompanied by GnRH immunostaining (red). **B**, **C** Bar graphs depict the MFI ratios of EGFP and GnRH across groups. (*n* = 3, Statistical analysis: Two-way ANOVA, Tukey’s post hoc test. *p < 0.05, **p < 0.01, ***p < 0.001). **D**. Representative bands show the phosphorylation levels of FAK, TGF-βR1, and Smad2, as well as their total protein levels, with GAPDH used as a loading control. **E**–**G** Quantification of the pFAK/FAK ratio (**E**), pTGF-βR1/TGF-βR1 ratio (**F**), and pSmad2/Smad2 ratio (**G**) (*n* = 4, Statistical analysis: Two-way ANOVA, Tukey’s post hoc test. *p < 0.05, ***p < 0.001)

We further investigated the role of Itgb1 in mediating the effects of EA on the hypothalamic GnRH–tanycyte unit. Figure 6 A shows the distribution of EGFP-labeled tanycytes (green) and GnRH axon terminals (red) in the ME and ARC. In the PCOS + Scramble group, tanycyte processes appeared sparse with weak fluorescence intensity, whereas GnRH-positive signals were notably stronger. EA treatment restored tanycyte process density and reduced GnRH aggregation. However, *Itgb1* knockdown disrupted the therapeutic effect of EA, resulting in impaired tanycyte processes and persistent GnRH axon aggregation (Fig. 6B, C). Western blot analysis further confirmed the involvement of the FAK/TGF-βR1/Smad2 signaling pathway (Fig. 6D). EA treatment significantly increased the phosphorylation of FAK (Fig. 6E), TGF-βR1 (Fig. 6F), and Smad2 (Fig. 6G) in the PCOS + EA + Scramble group, but these effects were diminished in the PCOS + EA + *Itgb1* knockdown group. These findings underscore the critical role of Itgb1 in mediating the regulatory effects of EA on tanycyte plasticity and GnRH axon dynamics. Itgb1 is essential for EA-mediated modulation of the hypothalamic GnRH–tanycyte unit, primarily through activation of the FAK/TGF-βR1/Smad2 signaling pathway.

## Discussion

**Fig. 7:**
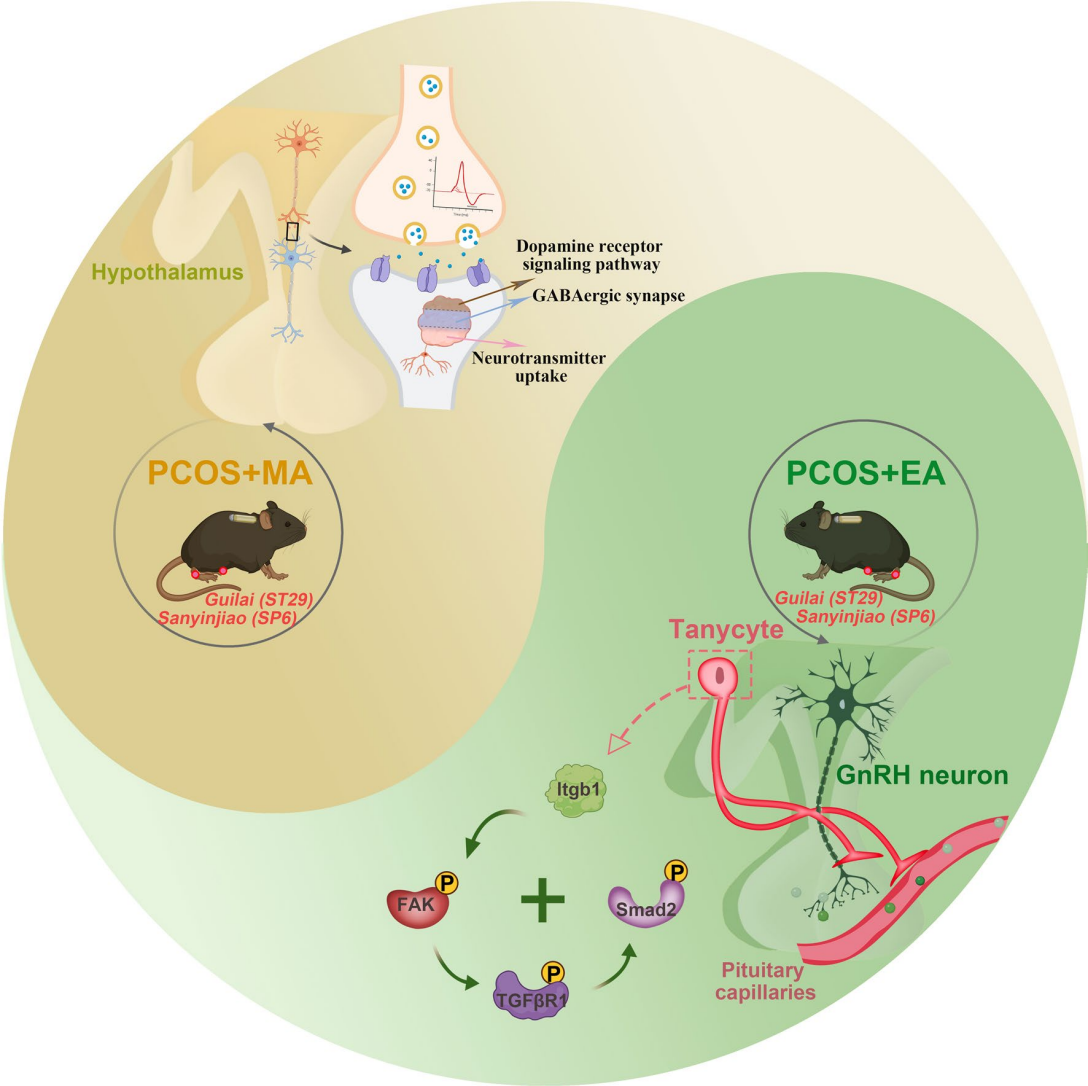
The diagram highlights the distinct mechanisms by which EA and MA modulate hypothalamic function in PCOS. EA primarily targets the GnRH–tanycyte unit, regulating Itgb1 and the FAK/TGF-βR1/Smad2 signaling pathway to maintain endocrine homeostasis. In contrast, MA predominantly influences neurotransmitter release and synaptic activity within the hypothalamus

In this study, we investigated the central mechanisms through which acupuncture alleviates PCOS symptoms using the tissue-clearing technique for the first time. EA distinctly enhances GnRH–tanycyte unit function by upregulating Itgb1, whereas MA potentially influences neuronal activity and hormone regulation. By identifying these specific central targets, this study provides valuable insights for optimizing acupuncture therapies (Fig. 7).

To elucidate the central mechanisms and differences between EA and MA, we focused on the hypothalamic GnRH networks with 3D imaging. Previous studies have shown that acupuncture regulates central β-endorphin production and influences the release of both GnRH and gonadotropins [36]. In this study, we provide the critical visualization of hypothalamic GnRH neuron changes induced by EA and MA. Compared with MA, EA more effectively reduces the aggregation of GnRH axonal terminals, highlighting its priority ability to modulate the hypothalamic microenvironment. Although MA also has the ability to influence the hypothalamic structure, its effects are less pronounced, suggesting that MA may operate through complementary but distinct therapeutic pathways. 3D imaging technology allows a broader examination of neuronal networks. This approach not only deepens our understanding of the mechanisms of acupuncture in treating PCOS but also paves the way for more targeted therapeutic interventions for other neuroendocrine disorders.

The relationship between GnRH neurons and hypothalamic glial cells has gained increasing attention in recent years. Astrocytes interact with the axon terminals of GnRH neurons and regulate their activity by releasing factors such as insulin-like growth factor and prostaglandin E2 [37–39]. Additionally, the number of hypothalamic microglia decreases before PCOS symptoms manifest [40]. The role of glial cells in shaping the GnRH neuronal microenvironment offers a potential avenue for central therapeutic strategies in PCOS. Tanycytes, specialized glial cells lining the third ventricle, have been shown to modulate hormone secretion through their interactions with GnRH neurons [13]. In PCOS, disruptions in the GnRH–tanycyte unit can alter GnRH pulsatility, resulting in the dysregulated release of LH and FSH [41]. Our findings suggest that EA counteracts the dysregulated hypothalamic signaling underlying PCOS pathology by rebalancing tanycyte process and neurotransmission. Conversely, MA appears to influence the GnRH–tanycyte unit more indirectly, as tanycyte morphology in the hypothalamic ARC and ME did not recover after MA treatment. This study highlights the GnRH–tanycyte unit as an important therapeutic target for PCOS, emphasizing the critical role of tanycytes in hypothalamic GnRH neurons regulation. The role of tanycytes on PCOS-related reproductive dysfunction is a novel perspective, shedding light on the targets of acupuncture in regulating HPOA dysfunction. This approach differs from studies focusing solely on the effects of HPOA on estrous cycle changes.

Research has identified several molecular pathways through which acupuncture affects hypothalamic regulation [42, 43]. However, the hypothalamic neural network is intricate and involves numerous molecular interactions, making the current research scope insufficiently comprehensive. In this study, we performed transcriptome analysis to identify and detect gene profile changes in the hypothalamus after EA and MA treatment. EA primarily targets pathways related to neuroglial plasticity and structural integrity within the hypothalamus, suggesting that its therapeutic effects are mediated by enhancing structural and signaling capacities in hypothalamic neurons and glial cells, particularly via the PI3K–Akt and TGF-beta signaling pathways. Maintaining hypothalamic neuroglial homeostasis is crucial for reproductive function, especially in individuals with PCOS, where disruption leads to abnormal GnRH pulsatility and hormonal imbalances [44, 45]. We found that EA activates the Itgb1 mediated FAK/TGFβR1/Smad2 signaling pathway, restoring the morphological plasticity of tanycyte processes and inhibiting the aggregation of GnRH axon terminals. Our findings revealed a specialized type of neuroglial interaction, suggesting that the GnRH–tanycyte unit may play a more specific role in the hypothalamic pathological mechanisms of PCOS and in the regulation of neuroendocrine function. Low-frequency EA activates somatic afferent nerves, A-δ fibers and C-fibers, which stimulate central nervous system inputs [46, 47]. This activation might restore neuro–glial connections and enhance the hypothalamic microenvironment. Conversely, MA appears to target pathways more directly associated with neuronal activity, neuropeptides and hormones, as reflected in its enrichment of processes such as dopamine neurotransmitter receptors, GABAergic synapses, and neuropeptide hormone activity. These findings suggest that MA may exert a more pronounced influence on neurotransmitter dynamics and endocrine functions, potentially stabilizing the HPOA through mechanisms distinct from those of EA.

The greater impact of EA on the plasticity of the GnRH–tanycyte unit suggests that EA may be a more effective treatment for addressing hypothalamic structural abnormalities in patients with PCOS. In contrast, the emphasis of MA on neuronal and hormonal regulation may make it better suited for normalizing hormone activity. Identifying these distinct and overlapping therapeutic targets is critical for advancing research and clinical practice, enabling more personalized and targeted treatment strategies. Patients with significant hypothalamic structural disruptions may benefit more from EA, whereas those with primarily functional hormone disturbances might respond better to MA.

Integrins, a family of transmembrane receptors, play crucial roles in cellular adhesion, signal transduction, and tissue remodeling [48, 49]. Among them, Itgb1 has emerged as a key player in PCOS research because of its involvement in regulating cell morphology and signaling pathways [50]. A novel finding of this study is the differential regulation of Itgb1 by EA and MA. EA significantly upregulates the expression of Itgb1, a protein that is vital for maintaining the homeostasis of GnRH–tanycyte unit. In contrast, MA has a milder effect on Itgb1, suggesting that its primary therapeutic effects may lie elsewhere, particularly in regulating neuronal activity and hormone secretion. This distinction highlights the greater therapeutic potential of EA in targeting the GnRH–tanycyte unit, which justifies the selection of EA for further investigation in transgenic mice. Although both EA and MA influence the integrin family, the stronger impact of EA on Itgb1 makes it a more powerful candidate for demonstrating how this protein contributes to the therapeutic effects of acupuncture. Moreover, as MA primarily regulates neural activity and hormone function, the knockdown of Itgb1 in tanycytes would likely provide less insight into the mechanisms of action of MA. One limitation of this study is the lack of further validation and specific interventions on the regulatory targets of MA, which should be explored in future research to fully understand the therapeutic targets of MA.

## Conclusion

Our research identified a critical mechanism by which acupuncture modulates the hypothalamic plasticity of the GnRH–tanycyte unit, and improves reproductive function in PCOS. Additionally, EA demonstrated a more pronounced effect than MA in restoring the GnRH–tanycyte unit. Our findings highlight Itgb1 as a potential therapeutic target for PCOS and propose that clinical treatment should utilize low-frequency EA at primary acupoints, supplemented with additional acupoints based on disease phenotype, combined with MA for enhanced efficacy.

## Supplementary Information

Supplementary material 1.

Supplementary material 2.

## Data Availability

The datasets used or analyzed throughout this study are available from the corresponding author upon reasonable request. The raw transcriptome sequencing data are available at the following link: https://bigd.big.ac.cn/gsa/browse/CRA018731.

## Abbreviations

PCOS: Polycystic ovary syndrome
GnRH: Gonadotropin-releasing hormone
EA: Electroacupuncture
MA: Manual acupuncture
HPOA: Hypothalamic-pituitary-ovarian axis
LH: Luteinizing hormone
AR: Androgen receptor
OGTT: Oral glucose tolerance test
ITT: Insulin tolerance test
HE: Hematoxylin and eosin
iDISCO: Immunolabeling-enabled three-dimensional imaging of solvent-cleared organs
DHT: Dihydrotestosterone
ARC: Arcuate nucleus
ME: Median eminence
DEGs: Differentially expressed genes
