# Supplementary material for: Distinct mechanisms of electroacupuncture and manual acupuncture in modulating hypothalamic GnRH–tanycyte unit function of polycystic ovary syndrome
Source: Chin Med. 2025 Feb 5;20:18. doi: 10.1186/s13020-025-01068-3 (PMC11796190; doi:10.1186/s13020-025-01068-3)
Supplement: Supplementary file 2 — Supplementary material 2. [file 13020_2025_1068_MOESM2_ESM.docx]

**Supporting Information**

**Distinct mechanisms of electroacupuncture and manual acupuncture in modulating hypothalamic GnRH–tanycyte unit function of polycystic ovary syndrome**

Yu Wang^1,2^, Yicong Wang^1,2^, Yuning Chen^1,2^, Wenhan Lu^3^, Xiaoyu Tong^1,2^, Jiajia Li^1,2^, Wenhao Gao^1,2^, Rui Huang^1,2^, Wei Hu^1,2^*, Yi Feng^1,2^*

^1^ Department of Integrative Medicine and Neurobiology, School of Basic Medical Sciences, State Key Laboratory of Medical Neurobiology and MOE Frontiers Center for Brain Science, Institutes of Brain Science, Fudan University, Shanghai, 200032, China.

^2^ Shanghai Key Laboratory of Acupuncture Mechanism and Acupoint Function, Shanghai Institute of Acupuncture and Moxibustion, Fudan University, Shanghai 200433, China.

^3^ Department of Ophthalmology & Visual Science, Eye & ENT Hospital, Shanghai Medical College, Fudan University, Shanghai, China.

* **Corresponding author:** Wei Hu E-mail: huwei_@fudan.edu.cn

Yi Feng E-mail: fengyi17@fudan.edu.cn


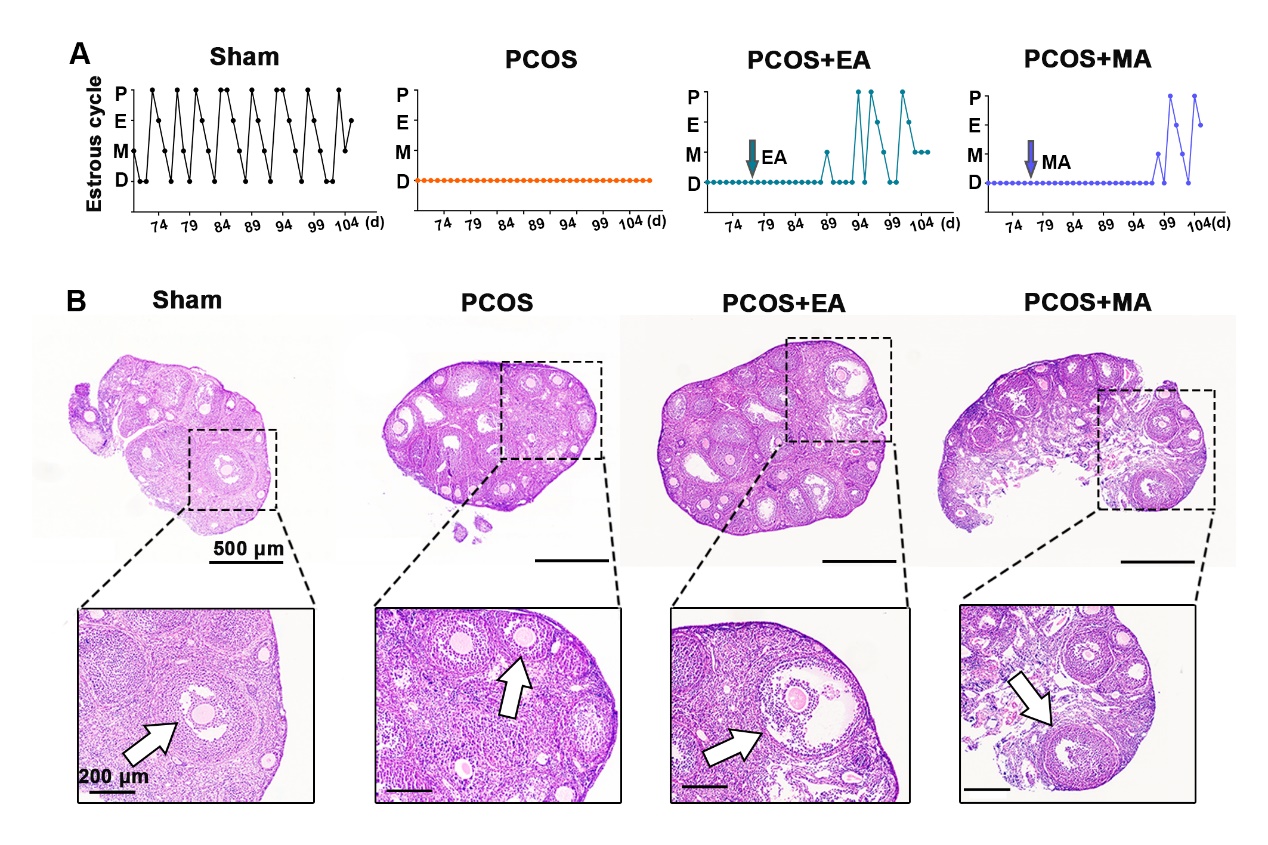


**Fig. S1** Acupuncture treatment restores ovarian reproductive function. **A.** Representative estrous cycle tracking over 30 days for the Sham, PCOS, PCOS+EA, and PCOS+MA groups (*n* = 5). **B**. Histological sections of ovarian tissue stained with hematoxylin and eosin, highlighting differences in follicular development across groups. Enlarged insets show representative follicles.


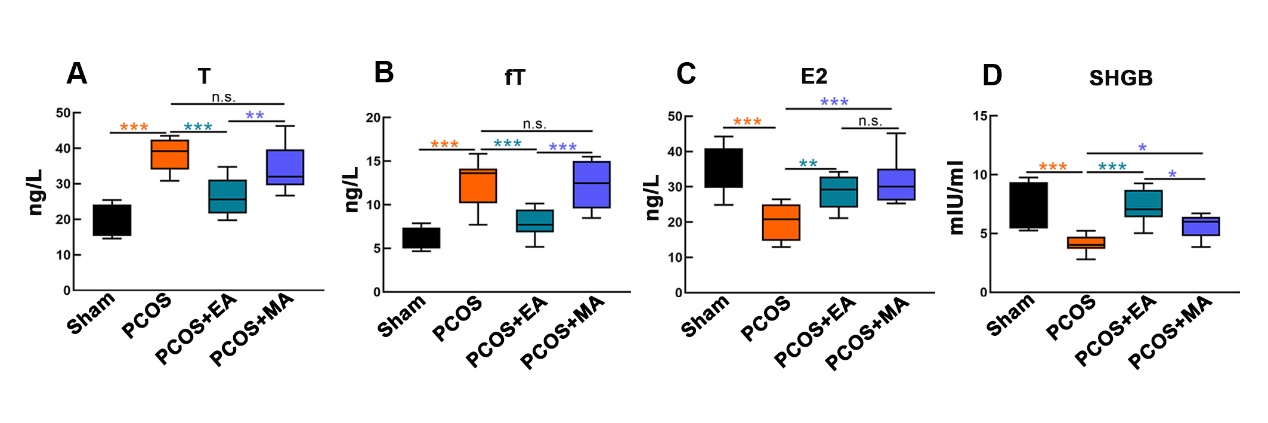
**Fig. S2 Acupuncture therapy restores serum sex hormone levels in PCOS-like mice**. (**A, B**) Box plots display the concentrations of serum testosterone (T) and free testosterone (fT) across four groups: Sham, PCOS, PCOS+EA, and PCOS+MA groups (*n* = 10, Statistical analysis: One-way ANOVA, (**A**) F_(3,36)_=25.37, P<0.0001; (**B**) F_(3,36)_=23.3, P<0.0001; Tukey’s post hoc test. ***p* < 0.01, ****p* < 0.001). (**C, D**) Box plots illustrate the serum concentrations of estradiol (E2) and sex hormone-binding globulin (SHGB) levels across the different groups (*n* = 10, Statistical analysis: One-way ANOVA, (**C**) F_(3,36)_=14.41, P<0.0001; (**D**) F_(3,36)_=15, P<0.0001; Tukey’s post hoc test. **p* < 0.05, ***p* < 0.01, ****p* < 0.001).


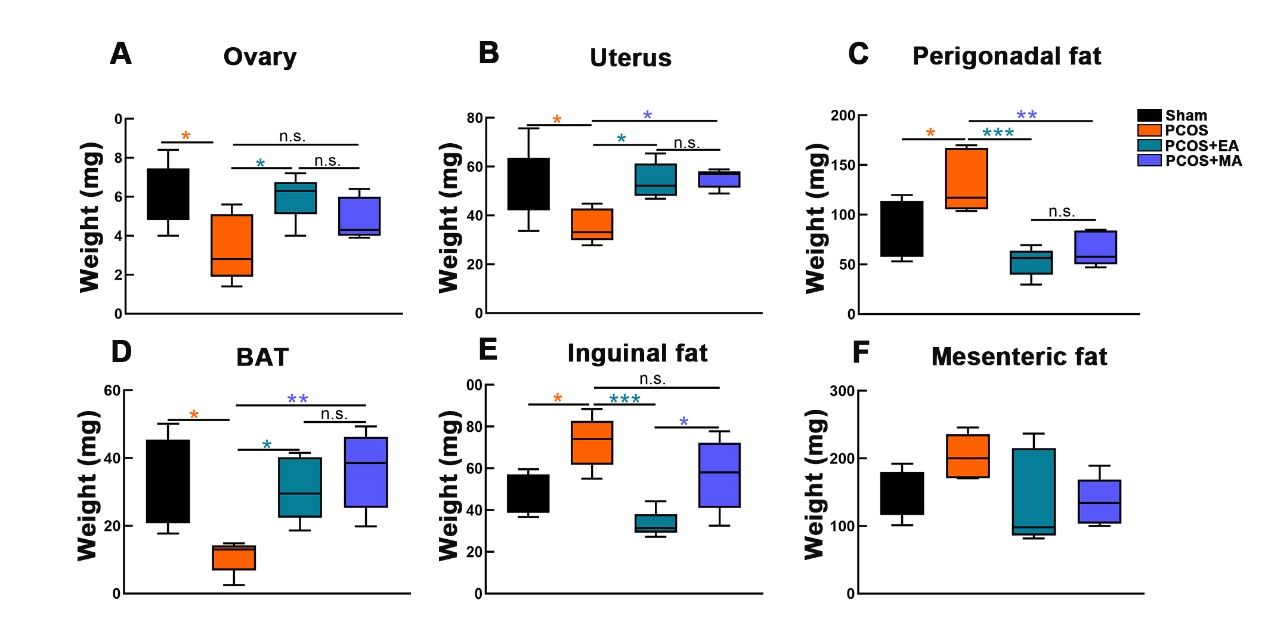
**Fig. S3 Acupuncture modulates gonadal and adipose tissue weights in PCOS-like mice.** This figure presents box plots comparing the weights of various tissues across four experimental groups: Sham, PCOS, PCOS+EA, and PCOS+MA. The panels display the following tissue weights: ovary (**A**), uterus (**B**), perigonadal fat (**C**), brown adipose tissue (BAT) (**D**), inguinal fat (**E**), and mesenteric fat (**F**). Each panel offers a comparison of tissue weights under different experimental conditions, highlighting variations in tissue mass among the groups (*n* = 5, Statistical analysis: One-way ANOVA, (**A**) F_(3,16)_=4.06, P=0.0253; (**B**) F_(3,16)_=10.35, P=0.0005; (**C**) F_(3,16)_=4.935, P=0.0130; (**D**) F_(3,16)_=6.483, P=0.0044; (**E**) F_(3,16)_=2.286, P=0.1178; (**F**) F_(3,16)_=9.457, P=0.0008; Tukey’s post hoc test. **p* < 0.05, ***p* < 0.01, ****p* < 0.001).


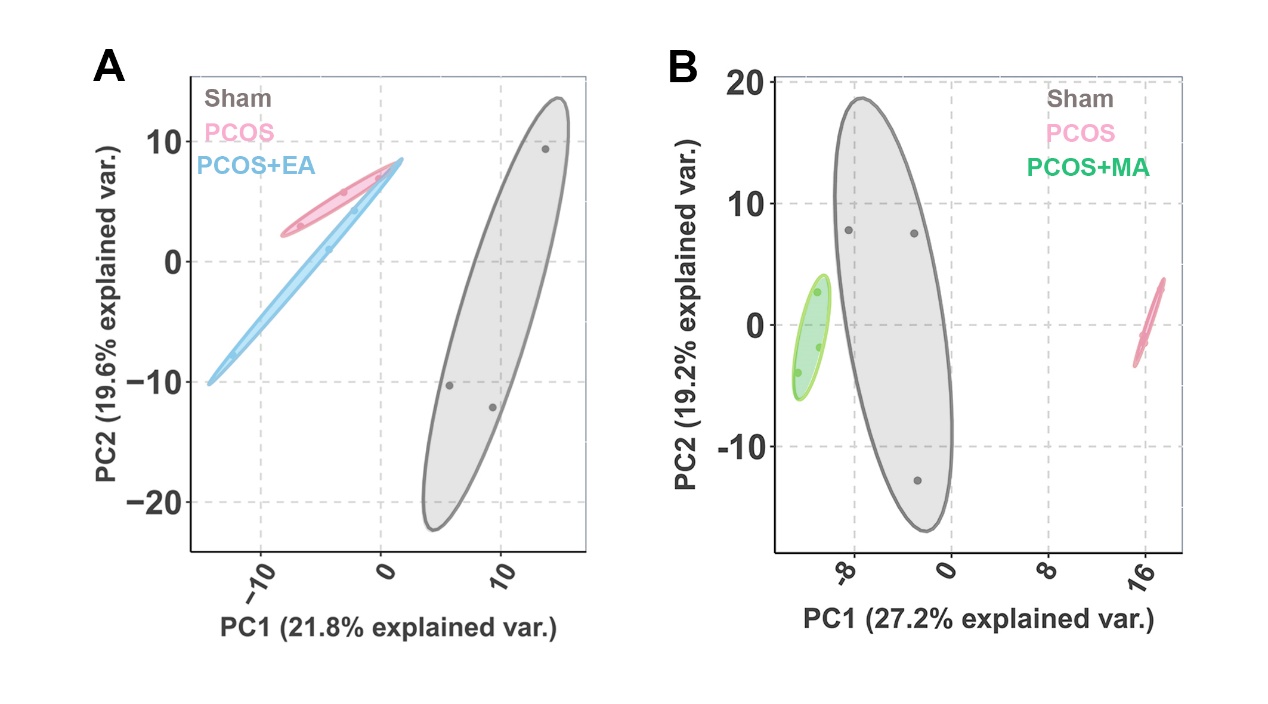


**Fig. S4 Principal component analysis (PCA) in hypothalamic samples. (A, B)** PCA plots illustrate the clustering of gene expression profiles among Sham, PCOS, PCOS+EA, and PCOS+MA groups. PC1 and PC2 represent the major components explaining variance in gene expression between the groups.


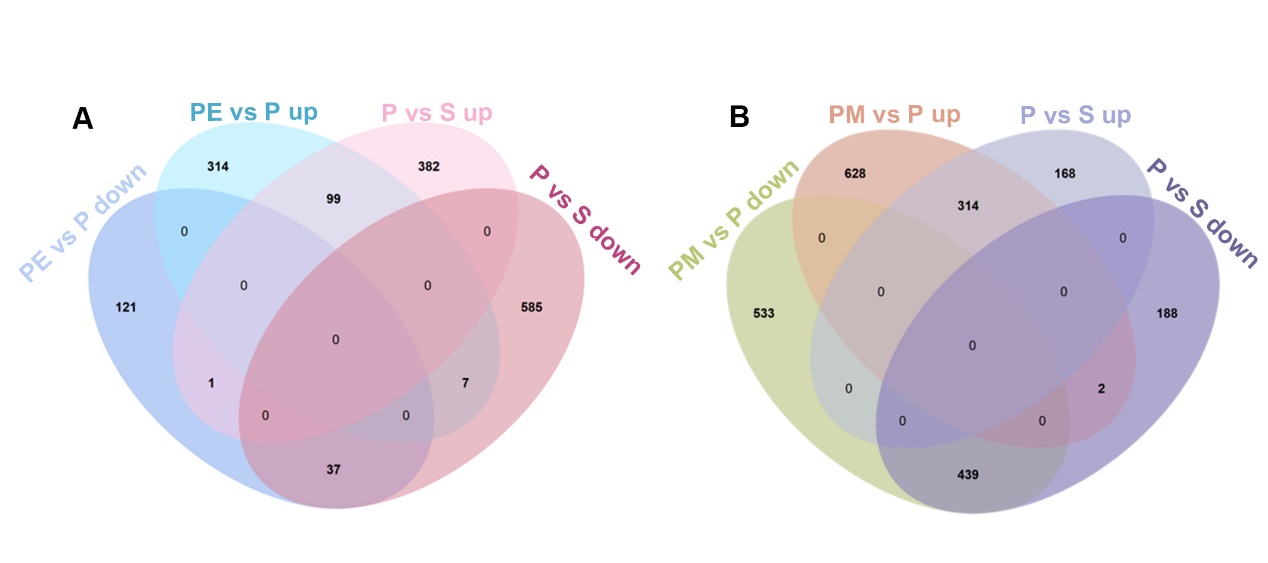
 **Fig. S5** **Venn diagrams depict the overlap of DEGs between various group comparisons.** **A**. The comparisons include PCOS+EA vs. PCOS upregulated genes (PE vs P up), PCOS+EA vs. PCOS downregulated genes (PE vs P down). **B.** PCOS+MA vs. PCOS upregulated genes (PM vs P up), and PCOS+MA vs. PCOS downregulated genes (PM vs P down), as well as the comparisons with Sham (P vs S up, P vs S down).


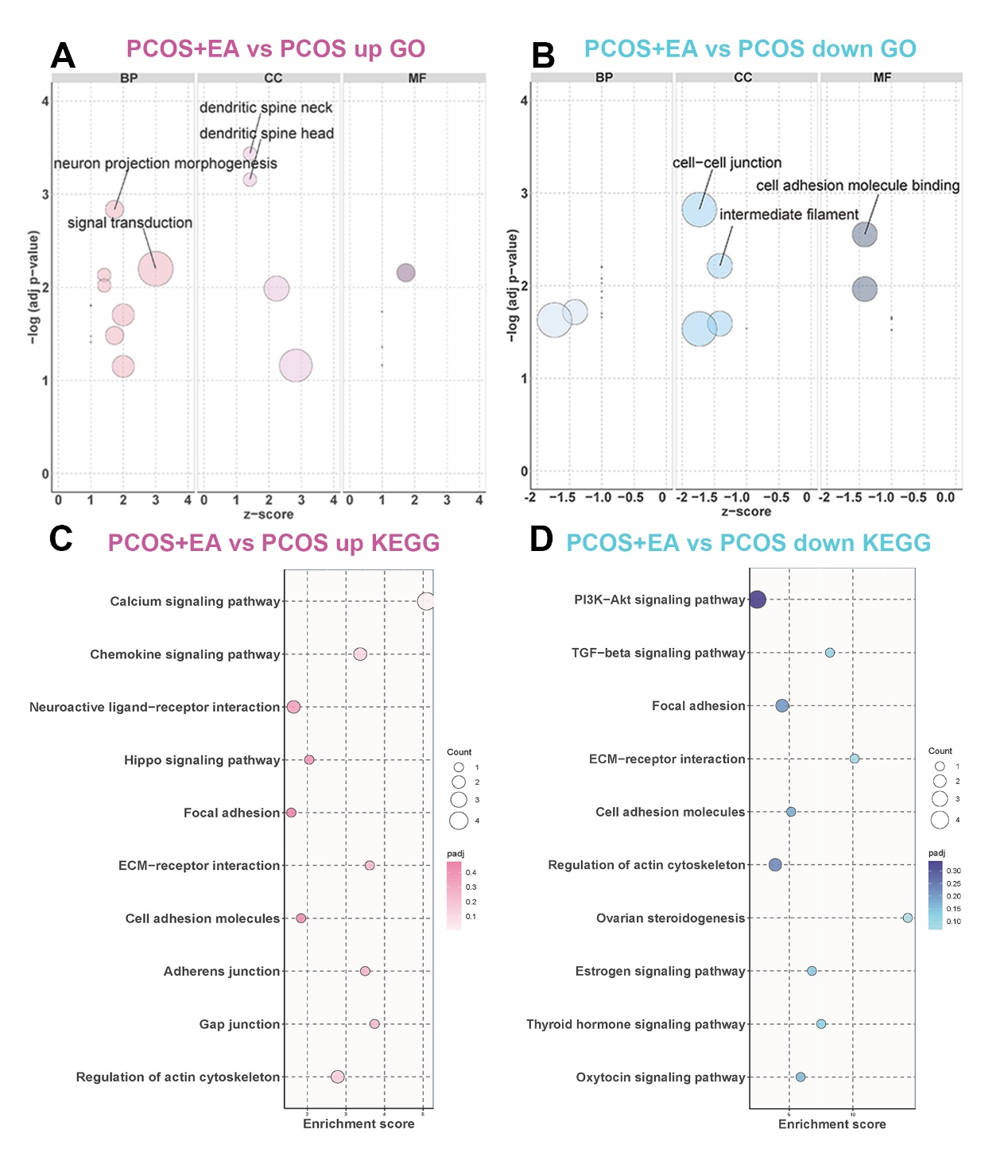


**Fig. S6 Functional enrichment analysis of DEGs between PCOS+EA and PCOS groups. A.** GO terms associated with upregulated genes in the PCOS+EA group, highlighting pathways related to neuronal morphogenesis and signal transduction. **B.** GO terms for downregulated genes, emphasizing cell adhesion and intermediate filament interactions. **C.** KEGG pathway analysis of upregulated genes, showing enrichment in calcium signaling, neuroactive ligand-receptor interaction, and focal adhesion pathways. **D.** KEGG pathway analysis of downregulated genes.


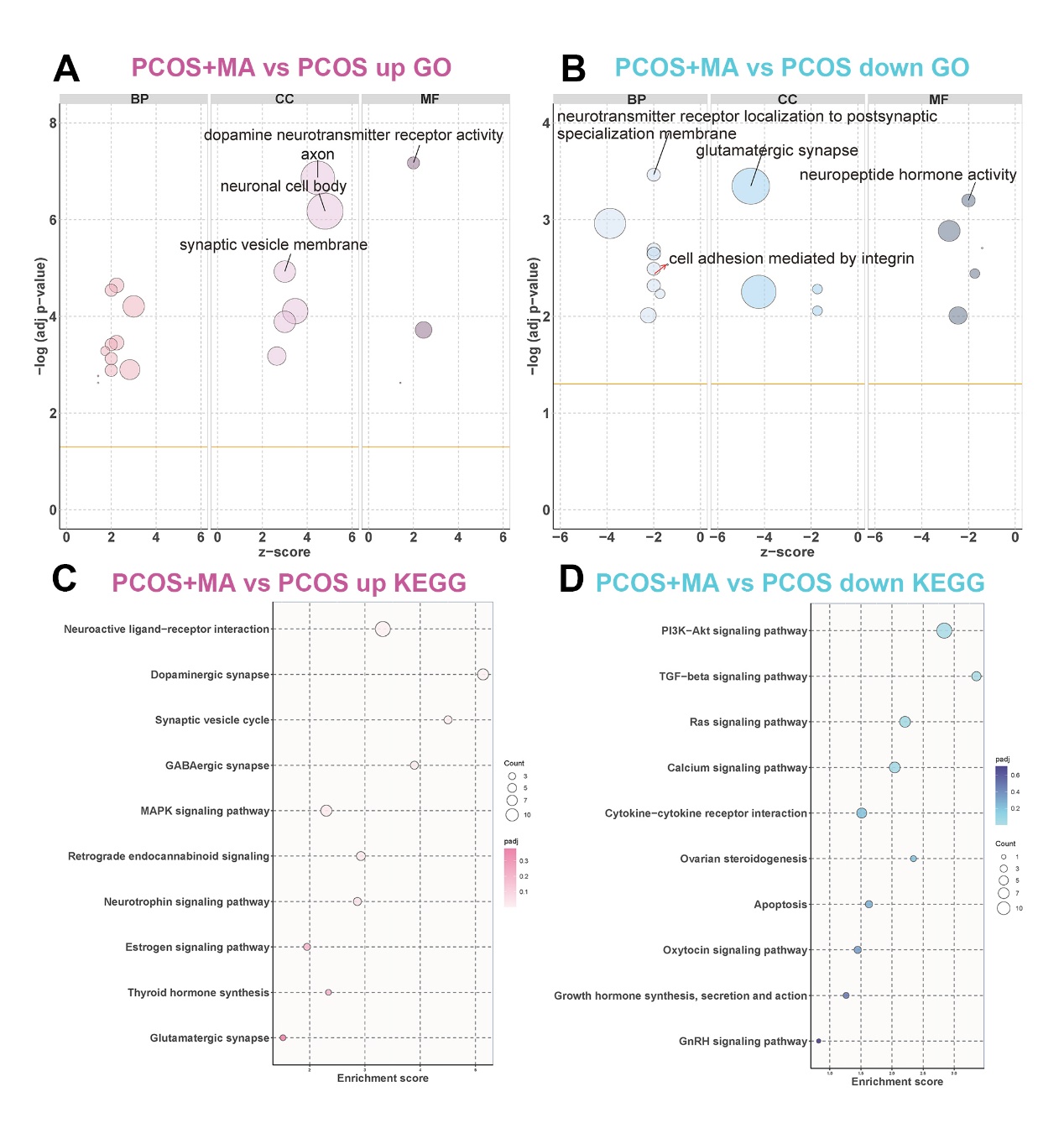


**Fig. S7 Functional enrichment analysis of DEGs between PCOS+MA and PCOS groups. A.** GO terms associated with upregulated genes in the PCOS+MA group, highlighting pathways related to neuronal cell body and synapse. **B.** GO terms for downregulated genes, emphasizing neuropeptides and neurotransmitters. **C.** KEGG pathway analysis of upregulated genes, showing enrichment in neuroactive ligand-receptor interaction, GABAergic synapse, and estrogen signaling pathways. **D.** KEGG pathway analysis of downregulated genes, identifying significant involvement of P13K-Akt signaling, TGF-beta signaling, and GnRH signaling pathways.
